# Nematic liquid crystalline elastomers are aeolotropic materials

**DOI:** 10.1098/rspa.2021.0259

**Published:** 2021-09

**Authors:** L. Angela Mihai, Haoran Wang, Johann Guilleminot, Alain Goriely

**Affiliations:** ^1^ School of Mathematics, Cardiff University, Senghennydd Road, Cardiff CF24 4AG, UK; ^2^ Department of Mechanical and Aerospace Engineering,Utah State University, Logan, UT 84322-4130, USA; ^3^ Department of Civil and Environmental Engineering, Duke University, Durham, NC 27708-0287, USA; ^4^ Mathematical Institute, University of Oxford, Woodstock Road, Oxford OX2 6GG, UK

**Keywords:** liquid crystals, elastomers, finite deformation, elastic moduli, anisotropy, molecular dynamics simulations

## Abstract

Continuum models describing ideal nematic solids are widely used in theoretical studies of liquid crystal elastomers. However, experiments on nematic elastomers show a type of anisotropic response that is not predicted by the ideal models. Therefore, their description requires an additional term coupling elastic and nematic responses, to account for aeolotropic effects. In order to better understand the observed elastic response of liquid crystal elastomers, we analyse theoretically and computationally different stretch and shear deformations. We then compare the elastic moduli in the infinitesimal elastic strain limit obtained from the molecular dynamics simulations with the ones derived theoretically, and show that they are better explained by including nematic order effects within the continuum framework.

## 1. Introduction

Liquid crystalline solids are responsive multifunctional materials that combine the flexibility of polymeric networks with the nematic order of liquid crystals [1,2]. Owing to their molecular architecture, they can exhibit dramatic spontaneous deformations and phase transitions, which are reversible and repeatable under heat, light, solvents and electric or magnetic fields [3–12]. However, their physical behaviour under combined mechanical loading and external stimuli still needs to be fully elucidated.

For ideal monodomain nematic elastomers, with the mesogens uniaxially aligned throughout the material, a continuum model is given by the so-called neoclassical strain-energy density introduced in [13–16]. This is a phenomenological strain-energy function that extends the neo-Hookean model for rubber [17], where the parameters are derived from macroscopic shape changes at small strain or through statistical averaging at microscopic scale [18,19]. Since elastic stresses dominate over Frank elasticity induced by the distortion of mesogens alignment [20–22], Frank energy [23,24] is generally neglected. Extensions to polydomains where every domain has the same strain-energy density as a monodomain are provided in [25,26]. These descriptions have been generalized by employing other hyperelastic models, such as Mooney–Rivlin, Gent and Ogden, which better capture the nonlinear elastic behaviour at large strains [27–29] (see [30,31] for molecular interpretations of the Mooney–Rivlin and Gent strain energies in rubber elasticity). Further generalizations can be found in [32,33]. Numerical studies of liquid crystal elastomers (LCEs) are presented, for example, in [33–35], where the finite-element method is used, and in [36,37], where molecular Monte Carlo simulations are employed.

Usually, when the elastic properties of a material are investigated, uniaxial deformations, which are easier to reproduce experimentally, are examined first [38–41]. For a purely elastic isotropic material, the shear modulus is then inferred from a universal relation between elastic moduli from the classical theory. To study the elastic responses of nematic monodomains, uniaxial deformations were assumed in [38] (see also [42]), where it was found that, if only the elastic energy was considered, then the stretch moduli in the direction parallel to the director and in a perpendicular direction were equal. However, experiments clearly show an aeolotropic effect; namely, the stretch moduli depend on the direction in which they are measured. To capture this experimentally observed response of the material, the nematic energy [43] was then also taken into account. Experimental results for monodomains where the tensile load formed different angles with the initial nematic director were reported in [39,44]. In [45], measurements of five independent elastic constants derived from three uniaxial tests, with the director parallel, perpendicular or at an angle of 45° relative to the loading direction, respectively, were obtained for a nematic monodomain treated as a classical transversely isotropic material. However, for many complex materials, shear deformations can reveal important additional mechanical effects, which may not be observed or inferred from uniaxial tests. In particular, to assess LCE materials, shear deformations with the direction of shear either parallel or perpendicular to the nematic director need to be considered independently of uniaxial stretches [46–52]. For example, on the one hand, it was found experimentally in [38] that, if $E_{∥}$ and *E*_⊥_ are the stretch moduli in a direction parallel or perpendicular to the nematic director, respectively, then $E_{∥}/E_{⊥}>1$ at low temperature, $E_{∥}/E_{⊥}<1$ at high temperature and $E_{∥}/E_{⊥}=1$ at the transition point. On the other hand, if $\mu_{∥}$ and *μ*_⊥_ denote the shear moduli in a direction parallel or perpendicular to the nematic director, respectively, then it was reported in [47,48,51] that $\mu_{∥}/\mu_{⊥}\approx 1$ in the isotropic phase and $\mu_{∥}/\mu_{⊥}<1$ at temperatures below that for the nematic–isotropic phase transition. Therefore, from a symmetry point of view, monodomain LCEs are transversely isotropic materials with five independent elastic constants and the distinguished direction given by the nematic director. However, despite the constitutive symmetry about the direction given by the nematic field, the mechanical responses of LCEs differ from the known elastic behaviours in traditional transversely isotropic elastic materials where, typically, $E_{∥}>E_{⊥}$ and$\mu_{∥}>\mu_{⊥}$ [53–56].

The aim of this study is to develop an explicit approach for the derivation of elastic moduli that captures the aeolotropy of liquid crystalline elastomers. This approach represents an extension of the general theoretical framework by which similar elastic moduli were obtained for hyperelastic materials [57]. In the case of nematic solids, these moduli include information about both the elasticity of the polymeric network and the mechanical responses of the liquid crystal molecules. In §2, we recall the neoclassical model for ideal nematic elastomers, with the isotropic phase at high temperature as the reference configuration [29,58–61], instead of the nematic phase at cross-linking [14–16,32,33,62,63]. Phenomenologically, this choice is motivated by the multiplicative decomposition of the effective deformation into an elastic distortion, followed by a natural stress-free shape change [64–67]. This multiplicative decomposition is similar to those found in the constitutive theories of thermoelasticity, elastoplasticity and growth [68,69] (see also [70,71]), but it is also different in the sense that the elastic deformation is directly applied to the reference state. The elastic stresses can then be used to study the final deformation where the stress-free geometrical change also plays a role [65]. In §3, we calculate the two *stretch moduli* under small elastic uniaxial tension and finite natural deformation when the nematic director is either parallel or perpendicular to the tensile direction, respectively. In §4, we further consider three shear deformations where the elastic component is a simple shear, while the nematic director is either parallel to the shear direction, perpendicular to the shear direction or perpendicular to the shear plane, respectively. When the elastic shear strain is small and the natural deformation is finite, we obtain effective *shear moduli* with the relative ratio equal to the natural anisotropy parameter of the nematic material. Note that we use the uniaxial and simple shear deformation, respectively, to describe the elastic contribution to the deformation rather than the overall deformation, which also contains a natural deformation component. To derive the elastic moduli, we then take the limit of small elastic strain, while the natural deformation remains finite. This enables us to rigorously adapt the elasticity theory to nematic elastomers (for a review on elastic moduli, see [57]). To account for the physical aeolotropy of real nematic solids, in §5, we extend the continuum model by incorporating a nematic energy, and show how the stretch and shear moduli corresponding to the ideal case are modified by the additional information. The parameters entering the LCE model characterize either the change of the microstructure due to nematic effects or the behaviour of the material in large natural deformations. Nevertheless, in the limit of small elastic deformations and for fixed nematic parameters, the system behaves indeed like a transverse isotropic material with five independent elastic constants. In physics, *aeolotropy* refers to materials exhibiting different properties depending on the direction in which they are measured, or simply defined by Lord Kelvin as ‘That which is different in different directions’ [72, p. 122]. While traditional anisotropic elastic materials also exhibit aeolotropy, we refer to nematic solids as *aeolotropic materials*, and reserve the characterization of *isotropic* or *anisotropic* for the elastic part of the energy. In §6, we present a molecular dynamics simulation of a nematic elastomer, and analyse its response under similar stretch and shear deformations as for the continuum model to illustrate the aeolotropic mechanical responses. In §§3–5, physical quantities are treated symbolically, and units of measure only appear in §6, where datasets are used to illustrate the theory. The final section containsconcluding remarks.

## 2. Prerequisites

The strain-energy density describing an ideal monodomain nematic liquid crystalline (NLC) solid takes the general form [64–67,73]

2.1 $$W^{\left(nc\right)}(\text{F},\text{n})=W(\text{A}),$$

where **F** represents the deformation gradient from the isotropic state, **n** is a unit vector, known as the *director*, for the orientation of the nematic field and *W*(**A**) denotes the strain-energy density of the isotropic polymer network, depending only on the (local) elastic deformation tensor **A**. The tensors **F** and **A** satisfy the following relation:

2.2 F=GA,

where

2.3 $$\text{G}=a^{1/3}\text{n}⊗\text{n}+a^{-\nu /3}(\text{I}-\text{n}⊗\text{n})$$

is the spontaneous deformation tensor describing a change of frame of reference from the isotropic to a nematic phase (e.g. [74]). In (2.3), *a* > 0 represents a temperature-dependent stretch parameter, *ν* is the optothermal analogue to the Poisson ratio [74] and relates responses in directions parallel or perpendicular to the director **n**, ⊗ denotes the tensor product of two vectors and **I** = diag(1, 1, 1) is the identity tensor. It is assumed here that *a* and *ν* are spatially independent. The ratio *r* = *a*^1/3^/*a*^−*ν*/3^ = *a*^(*ν*+1)/3^ represents the anisotropy parameter, which, in an ideal nematic solid, is the same in all directions. In the nematic phase, both the cases with *r* > 1 (prolate molecules) and *r* < 1 (oblate molecules) are possible, while when *r* = 1 the energy function reduces to that of an isotropic hyperelastic material. Nematic elastomers have *ν* = 1/2, while for nematic glasses *ν* ∈ (1/2, 2) [10,75]. Natural strains in NLC glasses are typically of up to 4%, whereas for NLC elastomers these may be up to 400%. The nematic director **n** is an observable (spatial) quantity. Denoting by **n**_0_ the reference orientation of the local director corresponding to the cross-linking state, **n** may differ from **n**_0_ both by a rotation and by a change in *r*. In nematic elastomers, which are weakly cross-linked, the director can rotate freely, and the material exhibits isotropic mechanical effects. In nematic glasses, which are densely cross-linked, the director **n** cannot rotate relative to the elastic matrix, but changes through convection due to elastic strain and satisfies [21,22,58,74,76]

2.4 $$\text{n}=\frac{\text{F}{\text{n}}_{0}}{|\text{F}{\text{n}}_{0}|}.$$

This constraint enables patterning of the director field at cross-linking and guarantees that the ‘written-in’ pattern remains virtually the same during natural shape changes [21,22,77].

For a hyperelastic material described by the strain-energy density *W* = *W*(**A**), the Cauchy stress tensor is equal to

2.5 $$\text{T}={\left(\det\text{A}\right)}^{-1}\frac{\partial W}{\partial \text{A}}{\text{A}}^{T}-p\text{I}=-p\text{I}+\beta_{1}\text{B}+\beta_{-1}{\text{B}}^{-1},$$

where the so-called hydrostatic pressure *p* denotes the Lagrange multiplier for the incompressibility constraint detA=1, *β*_1_ = 2∂*W*/∂*I*_1_ and *β*_−1_ = −2∂*W*/∂*I*_2_ are material parameters, **B** = **AA**^*T*^ is the left Cauchy–Green elastic deformation tensor and *I*_1_, *I*_2_ are its first two principal invariants (*I*_3_ = 1 owing to incompressibility). The corresponding first Piola–Kirchhoff stress tensor is equal to

2.6 P=TCof(A),

where $\mathrm{Cof}(\text{A})=(\det\text{A}){\text{A}}^{-T}$. For an NLC solid characterized by the strain-energy density given by (2.1), the stress tensors when the director is ‘free’ to rotate relative to the elastic matrix and when the nematic director is ‘frozen’ and satisfies condition (2.4) are presented in appendix A (see also [65]).

In the next sections, we obtain two *stretch moduli* under small elastic uniaxial tension and three nonlinear *shear moduli* under elastic simple shear deformations, respectively, which combine elastic and nematic effects. In our calculations, the nematic director is ‘frozen’, but the case where the director is ‘free’ can be treated similarly, provided that the elastic deformation is small. In particular, when the director is free to rotate and a tensile force is applied perpendicular to the director, experimental results show that there is a range of strains, up to 10% (e.g. [78,79]), before the director rotates in response to the applied force. However, the local nematic order might be altered. We further note that, in finite elasticity, the strain-energy density *W*(**A**) and the stress relationship (2.5) characterize an isotropic material. Yet, the response of a nematic solid also depends on the director orientation. For instance, we show that the ideal model exhibits an anisotropic response under stretch if the natural deformation is finite. There are, in fact, two possible contributions to aeolotropy. First, in the ideal case, the elastic energy of the system is isotropic but the full energy depends on the orientation of the nematic field as well [59]. Second, there is a component of the energy that depends directly on the nematic order through the so-called **Q**-tensor [23]. In this case, we need to extend the discussion by including this nematic energy density as developed later in the paper.

## 3. Stretch moduli

The stretch modulus of a homogeneous isotropic elastic material is obtained under uniaxial tension with the gradient tensor in a Cartesian system of coordinates taking the form [57]

3.1 $$\text{A}=\left[\begin{array}{ccc} 1/\sqrt{\lambda } & 0 & 0\\ 0 & \lambda & 0\\ 0 & 0 & 1/\sqrt{\lambda } \end{array}\right],$$

where *λ* > 1 is the stretch ratio in the direction of the applied tensile force. Assuming that the only non-zero component of the associated first Piola–Kirchhoff stress, given by (2.6), is in the tensile direction, i.e. *P*_22_ > 0, it follows that

3.2 $$P_{22}=\left(\beta_{1}-\frac{\beta_{-1}}{\lambda }\right)\left(\lambda -\frac{1}{\lambda^{2}}\right).$$

The Young modulus at small strain is then defined as [57]

3.3 $$E=\lim_{\lambda_{1}\to 1}\frac{P_{22}}{A_{22}-1}=\lim_{\lambda \to 1}\frac{P_{22}}{\lambda -1}=3\lim_{\lambda \to 1}(\beta_{1}-\beta_{-1})=3\mu ,$$

where *P*_22_ is the tensile first Piola–Kirchhoff stress given by (3.2), *λ* − 1 is the corresponding tensile strain (for different definitions of an elastic strain, see [57]) and *μ* = lim _*λ*→1_(*β*_1_ − *β*_−1_) is the shear modulus at small strain.

To derive stretch moduli for the nematic material, we apply the tensile force in a direction that is either parallel or perpendicular to the reference nematic director [45]. In each case, we assume an overall deformation where the elastic component is a uniaxial tension with the deformation tensor given by (3.1). Then, we calculate the stretch moduli by taking the ratio between the first Piola–Kirchhoff tensile stress and the associated strain in the limit of *small elastic tensile strain*, while the natural deformation remains finite.

### (a) Nematic director parallel or perpendicular to the tensile direction

**Figure 1.**
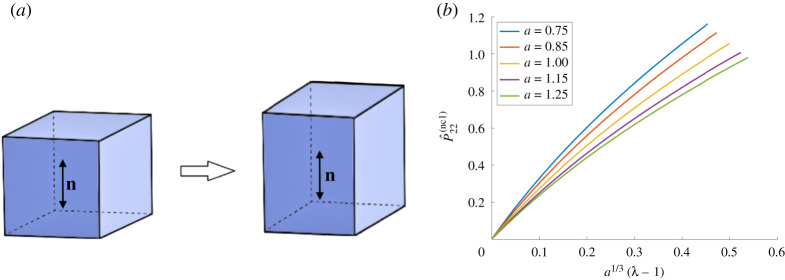
(*a*) Nematic material with the director parallel to the applied tensile force, and (*b*) the effect of varying the parameter *a* on the first Piola–Kirchhoff tensile stress of an ideal material when *μ* = 1 and *ν* = 1/2. (Online version in colour.)

When the tensile force is acting in the second Cartesian direction and the reference director is parallel to the tensile force, the nematic director in the current configuration and the associated spontaneous deformation tensor take the following form, respectively (figure 1*a*):

3.4 $${\text{n}}^{\left(1\right)}=\left[\begin{array}{c} 0\\ 1\\ 0 \end{array}\right],\;{\text{G}}^{\left(1\right)}=\left[\begin{array}{ccc} a^{-\nu /3} & 0 & 0\\ 0 & a^{1/3} & 0\\ 0 & 0 & a^{-\nu /3} \end{array}\right].$$

**Figure 2.**
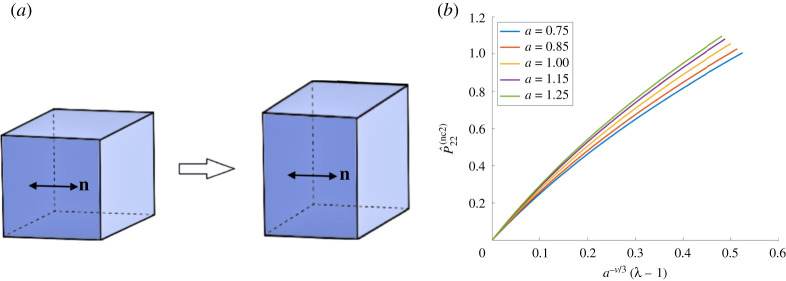
(*a*) Nematic material with the director perpendicular to the applied tensile force, and (*b*) the effect of varying the parameter *a* on the first Piola–Kirchhoff tensile stress of an ideal material when *μ* = 1 and *ν* = 1/2. (Online version in colour.)

When the reference director is perpendicular to the tensile force, the nematic director in the current configuration and the associated spontaneous deformation tensor are, respectively (figure 2*a*),

3.5 $${\text{n}}^{\left(2\right)}=\left[\begin{array}{c} 1\\ 0\\ 0 \end{array}\right],\;{\text{G}}^{\left(2\right)}=\left[\begin{array}{ccc} a^{1/3} & 0 & 0\\ 0 & a^{-\nu /3} & 0\\ 0 & 0 & a^{-\nu /3} \end{array}\right].$$

Assuming that the elastic deformation tensor **A** is of the form given by (3.1), for the deformation gradient **F** and the associated first Piola–Kirchhoff stress, the principal components in the direction of the tensile load are, respectively,

3.6 $$F_{22}^{\left(1\right)}=\lambda a^{1/3},\;{\hat{P}}_{22}^{\left(\mathrm{nc}1\right)}=a^{-1/3}P_{22}$$

and

3.7 $$F_{22}^{\left(2\right)}=\lambda a^{-\nu /3},\;{\hat{P}}_{22}^{\left(\mathrm{nc}2\right)}=a^{\nu /3}P_{22},$$

where *P*_22_ is the first Piola–Kirchhoff stress given by (3.2). These are illustrated in figures 1*b* and 2*b*, respectively, for *μ* = 1 and *ν* = 1/2. We define the following stretch moduli for the nematic material under the above two deformations, respectively:

3.8 $$E^{\left(1\right)}=\lim_{\lambda \to 1}\frac{{\hat{P}}_{22}^{\left(\mathrm{nc}1\right)}}{F_{22}^{\left(1\right)}-a^{1/3}}=\lim_{\lambda \to 1}\frac{a^{-1/3}P_{22}}{a^{1/3}(\lambda -1)}=a^{-2/3}\lim_{\lambda \to 1}\frac{P_{22}}{\lambda -1}=Ea^{-2/3}$$

and

3.9 $$E^{\left(2\right)}=\lim_{\lambda \to 1}\frac{{\hat{P}}_{22}^{\left(\mathrm{nc}2\right)}}{F_{22}^{\left(2\right)}-a^{-\nu /3}}=\lim_{\lambda \to 1}\frac{a^{\nu /3}P_{22}}{a^{-\nu /3}(\lambda -1)}=a^{2\nu /3}\lim_{\lambda \to 1}\frac{P_{22}}{\lambda -1}=Ea^{2\nu /3},$$

where *E* = 3*μ* is Young’s modulus defined by (3.3). Therefore, the stretch moduli given by (3.8) and (3.9) satisfy the following relation:

3.10 $$\frac{E^{\left(2\right)}}{E^{\left(1\right)}}=a^{2(\nu +1)/3}=r^{2}.$$

We infer that: if *r* > 1, then *E*^(1)^ < *E*^(2)^; if *r* < 1, then *E*^(1)^ > *E*^(2)^; if *r* = 1, then *E*^(1)^ = *E*^(2)^ = *E*.

## 4. Shear moduli

To obtain the shear modulus of a homogeneous isotropic elastic material, a standard deformation is the simple shear, with the following gradient tensor in a Cartesian system ofcoordinates [57]:

4.1 $$\text{A}=\left[\begin{array}{ccc} 1 & k & 0\\ 0 & 1 & 0\\ 0 & 0 & 1 \end{array}\right],$$

where *k* > 0 is the shear parameter. The non-zero components of the associated Cauchy stress tensor, given by (2.5), are

4.2 $$T_{11}=\beta_{1}k^{2},\;T_{22}=\beta_{-1}k^{2},\;T_{12}=k(\beta_{1}-\beta_{-1}),$$

and the non-zero components of the corresponding first Piola–Kirchhoff stress tensor, given by (2.6), are

4.3 $$P_{11}=T_{11}-kT_{12},\;P_{22}=T_{22},\;P_{12}=T_{12},\;P_{21}=-kT_{12}.$$

The shear modulus at small shear is then obtained as follows [57]:

4.4 $$\mu =\lim_{k\to 0}\frac{P_{12}}{A_{12}}=\lim_{k\to 0}\frac{P_{12}}{k}=\lim_{k\to 0}(\beta_{1}-\beta_{-1}).$$

To obtain suitable shear moduli for the nematic solid, we assume an overall deformation where the elastic component is a simple shear with the deformation tensor given by (4.1). In general, one cannot easily separate the individual contributions to the deformation gradient given by (2.2). However, for the three shear configurations adopted here, it is possible to write these components explicitly. Then, in each case, we can calculate the shear modulus by taking the ratio between the first Piola–Kirchhoff shear stress and the corresponding shear strain in the limit of *small elastic shearing strains*, while the natural deformation remains finite.

### (a) Nematic director parallel to the shear direction

**Figure 3.**
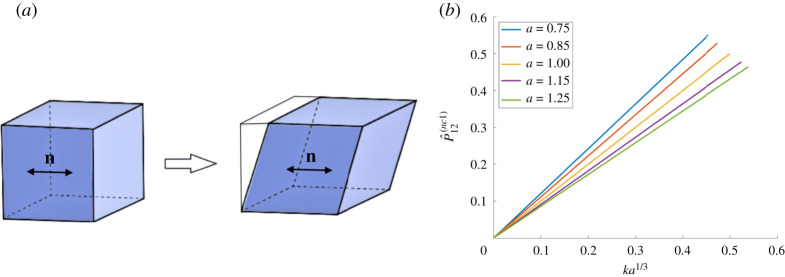
(*a*) Nematic material with the director parallel to the applied shear force, and (*b*) the effect of varying the parameter *a* on the first Piola–Kirchhoff shear stress when *μ* = 1 and *ν* = 1/2. (Online version in colour.)

When the reference nematic director ${\text{n}}_{0}^{\left(1\right)}=[1,0,0{]}^{T}$ is parallel to the direction of applied shear force, in the current configuration, we have (figure 3*a*)

4.5 $$\begin{array}{rl} & {\text{n}}^{\left(1\right)}=\left[\begin{array}{c} 1\\ 0\\ 0 \end{array}\right],\\ & {\text{G}}^{\left(1\right)}=\left[\begin{array}{ccc} a^{1/3} & 0 & 0\\ 0 & a^{-\nu /3} & 0\\ 0 & 0 & a^{-\nu /3} \end{array}\right]. \end{array}\}$$

By (2.2) and (A4), the corresponding shear strain and first Piola–Kirchhoff shear stress for the nematic solid are, respectively,

4.6 $$F_{12}^{\left(1\right)}=ka^{1/3}\;\text{and}\;{\hat{P}}_{12}^{\left(\mathrm{nc}1\right)}=a^{-1/3}P_{12},$$

where *P*_12_ is the shear component of the elastic first Piola–Kirchhoff stress given by (4.3). These are represented in figure 3*b*, for *μ* = 1 and *ν* = 1/2. We now define the shear modulus for the nematic material at small shear as follows:

4.7 $$\mu^{\left(1\right)}=\lim_{k\to 0}\frac{{\hat{P}}_{12}^{\left(\mathrm{nc}1\right)}}{F_{12}^{\left(1\right)}}=\lim_{k\to 0}\frac{P_{12}}{k}a^{-2/3}=\lim_{k\to 0}(\beta_{1}-\beta_{-1})a^{-2/3}=\mu a^{-2/3}.$$

### (b) Nematic director perpendicular to the shear direction

**Figure 4.**
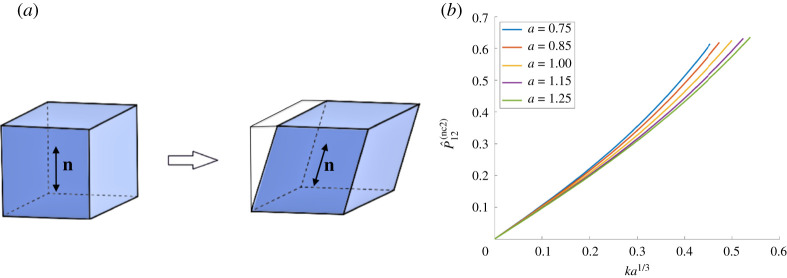
(*a*) Nematic material with the director perpendicular to the applied shear force, and (*b*) the effect of varying the parameter *a* on the first Piola–Kirchhoff shear stress when *μ* = 1 and *ν* = 1/2. (Online version in colour.)

When the reference nematic director ${\text{n}}_{0}^{\left(2\right)}=[0,1,0{]}^{T}$ is perpendicular to the direction of shear, we obtain in the current configuration (figure 4*a*)

4.8 $$\begin{array}{rl} & {\text{n}}^{\left(2\right)}=\frac{1}{\sqrt{k^{2}+1}}\left[\begin{array}{c} k\\ 1\\ 0 \end{array}\right]\\ \;\text{and}\; & {\text{G}}^{\left(2\right)}=\frac{1}{k^{2}+1}\left[\begin{array}{ccc} k^{2}a^{1/3}+a^{-\nu /3} & k(a^{1/3}-a^{-\nu /3}) & 0\\ k(a^{1/3}-a^{-\nu /3}) & k^{2}a^{-\nu /3}+a^{1/3} & 0\\ 0 & 0 & a^{-\nu /3}(k^{2}+1) \end{array}\right]. \end{array}\}$$

For sufficiently small values of *k*, the corresponding shear strain and first Piola–Kirchhoff shear stress of the nematic material take the form

4.9 $$F_{12}^{\left(2\right)}=ka^{1/3},\;{\hat{P}}_{12}^{\left(\mathrm{nc}2\right)}=a^{(\nu -1)/3}(G_{22}^{\left(2\right)}P_{12}-G_{12}^{\left(2\right)}P_{22}),$$

with the components *P*_12_ and *P*_22_ of the elastic first Piola–Kirchhoff stress given by (4.3). These are illustrated in figure 4*b*, for *μ* = 1 and *ν* = 1/2. In this case, the associated shear modulus at small shear is equal to

4.10 $$\mu^{\left(2\right)}=\lim_{k\to 0}\frac{{\hat{P}}_{12}^{\left(\mathrm{nc}2\right)}}{F_{12}^{\left(2\right)}}=\lim_{k\to 0}(\beta_{1}-\beta_{-1})a^{(\nu -1)/3}=\mu a^{(\nu -1)/3}.$$

### (c) Nematic director perpendicular to the shear plane

**Figure 5.**
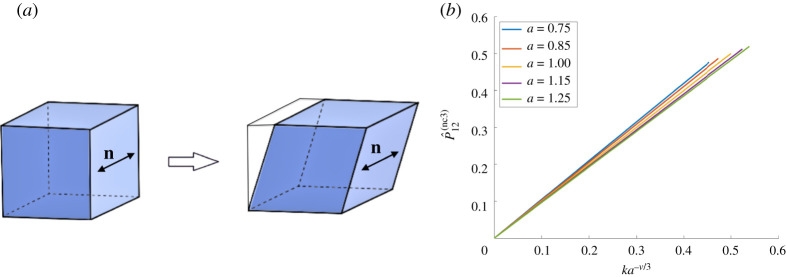
(*a*) Nematic material with the director perpendicular to the shear plane, and (*b*) the effect of varying the parameter *a* on the first Piola–Kirchhoff shear stress when *μ* = 1 and *ν* = 1/2. (Online version in colour.)

When the reference nematic director ${\text{n}}_{0}^{\left(3\right)}=[0,0,1{]}^{T}$ is perpendicular to the shear plane, we have (figure 5*a*)

4.11 $${\text{n}}^{\left(3\right)}=\left[\begin{array}{c} 0\\ 0\\ 1 \end{array}\right],\;{\text{G}}^{\left(3\right)}=\left[\begin{array}{ccc} a^{-\nu /3} & 0 & 0\\ 0 & a^{-\nu /3} & 0\\ 0 & 0 & a^{1/3} \end{array}\right].$$

By (2.2) and (A4), the corresponding shear strain and first Piola–Kirchhoff shear stress for the nematic solid are, respectively,

4.12 $$F_{12}^{\left(3\right)}=ka^{-\nu /3}\;\text{and}\;{\hat{P}}_{12}^{\left(\mathrm{nc}3\right)}=a^{\nu /3}P_{12},$$

where *P*_12_ is the shear component of the elastic first Piola–Kirchhoff stress given by (4.3). These are represented in figure 5*b*, for *μ* = 1 and *ν* = 1/2. We now define the shear modulus for the nematic material at small shear as follows:

4.13 $$\mu^{\left(3\right)}=\lim_{k\to 0}\frac{{\hat{P}}_{12}^{\left(\mathrm{nc}3\right)}}{F_{12}^{\left(3\right)}}=\lim_{k\to 0}\frac{P_{12}}{k}a^{2\nu /3}=\lim_{k\to 0}(\beta_{1}-\beta_{-1})a^{2\nu /3}=\mu a^{2\nu /3}.$$

A comparison of the shear moduli given by (4.7), (4.10) and (4.13) implies

4.14 $$\frac{\mu^{\left(2\right)}}{\mu^{\left(1\right)}}=\frac{\mu^{\left(3\right)}}{\mu^{\left(2\right)}}=a^{(\nu +1)/3}=r.$$

We conclude that: if *r* > 1, then *μ*^(1)^ < *μ*^(2)^ < *μ*^(3)^; if *r* < 1, then *μ*^(1)^ > *μ*^(2)^ > *μ*^(3)^; if *r* = 1, then *μ*^(1)^ = *μ*^(2)^ = *μ*^(3)^ = *μ*.

## 5. Contribution of the nematic free energy

The results of §§3 and 4 imply that, for an ideal nematic material, the effective shear and stretch moduli respect the same inequalities (e.g. if *r* > 1, then we have both *E*^(1)^ < *E*^(2)^ and *μ*^(1)^ < *μ*^(2)^ < *μ*^(3)^, and so on). However, numerous experimental results have demonstrated that there are significant differences between the behaviour of real nematic solids and that of ideal nematic materials analysed in the previous sections [38,42,45]. In particular, it was found that the ideal behaviour of the stretch and shear moduli ratio does not match experimental results. We therefore extend the strain-energy function by taking into account the nematic free-energy density, in addition to the isotropic strain-energy density *W*^(nc)^ = *W*^(nc)^(**F**, **n**), given by (2.1), as

5.1 $$W^{\left(\text{lce}\right)}=W^{\left(\text{nc}\right)}+W_{n},$$

where *W*_*n*_ is equal to [38,43] (see also [19], ch. 2)

5.2 $$W_{n}(\text{Q})=\frac{1}{3}A\mathrm{tr}(\text{Q}\text{Q})-\frac{4}{9}B\mathrm{tr}(\text{Q}\text{Q}\text{Q})+\frac{2}{9}C\mathrm{tr}(\text{Q}\text{Q}\text{Q}\text{Q})+\cdots ,$$

with **Q** the order parameter tensor. This macroscopic tensor parameter is used to describe the orientational order in nematic liquid crystals [23] (see also [80]). For incompressible nematic elastomers subjected to uniaxial stretches, the contribution given by (5.2) to the total strain-energy density described by (5.1) was originally analysed in [38]. Following a similar approach, we restrict our attention to the one-term strain-energy density of the form (2.1) (see also [58]),

5.3 $$W^{\left(\text{nc}\right)}(\text{F},\text{n})=\frac{\mu }{2}\mathrm{tr}({\text{F}}^{T}{\text{G}}^{-2}\text{F}),$$

where ‘tr’ denotes the trace operator and *μ* > 0 is the elastic shear modulus at small strain. This strain-energy density can be expressed equivalently as follows [14]:

5.4 $${\tilde{W}}^{\left(\text{nc}\right)}(\bar{\text{F}},\text{n})=\frac{\mu }{2}\mathrm{tr}({\text{G}}_{0}^{2}{\bar{\text{F}}}^{T}{\text{G}}^{-2}\bar{\text{F}}),$$

where ${\text{G}}_{0}=\mathrm{diag}({\left(l_{∥}^{0}\right)}^{1/2},{\left(l_{⊥}^{0}\right)}^{1/2},{\left(l_{⊥}^{0}\right)}^{1/2})$ is the natural deformation tensor corresponding to the cross-linking state (with eigenvalues ${\left(l_{∥}^{0}\right)}^{1/2}$ and ${\left(l_{⊥}^{0}\right)}^{1/2}$, ∥ denoting the direction parallel to the director and ⊥ indicating an orthogonal direction), $\text{G}=\mathrm{diag}(l_{1}^{1/2},l_{2}^{1/2},l_{3}^{1/2})$ is the natural deformation tensor in the current configuration (with eigenvalues $l_{1}^{1/2}$, $l_{2}^{1/2}$, $l_{3}^{1/2}$) and $\bar{\text{F}}$ satisfies the relation $\text{F}=\bar{\text{F}}{\text{G}}_{0}$ [29]. When the order parameter tensor takes the form **Q** = diag( − (*Q* − *b*)/2, − (*Q* + *b*)/2, *Q*), where *Q* and *b* are scalar values (see also [19], §2.2), we consider the nematic energy described by (5.2) and approximate it by [38]

5.5 $${\tilde{W}}_{n}(Q,b)=\frac{1}{2}AQ^{2}-\frac{1}{3}BQ^{3}+\frac{1}{4}CQ^{4}+\cdots +\frac{1}{6}(A+2BQ+CQ^{2}+\cdots )b^{2}+\cdots .$$

We therefore approximate the total strain-energy density defined by (5.1) as

5.6 $${\tilde{W}}^{\left(\text{lce}\right)}={\tilde{W}}^{\left(\text{nc}\right)}+{\tilde{W}}_{n},$$

where ${\tilde{W}}^{\left(\text{nc}\right)}={\tilde{W}}^{\left(\text{nc}\right)}(\bar{\text{F}},\text{n})$ and ${\tilde{W}}_{n}={\tilde{W}}_{n}(Q,b)$ are given by (5.4) and (5.5), respectively. For the components of **G**^2^, the first-order approximation of the Taylor expansions about the backbone order parameters in the initial state (*Q*, *b*) = (*Q*_0_, 0) are [38]

5.7 $$l_{1}\approx l_{∥}^{0}\left(1+\frac{l_{∥,Q}}{l_{∥}^{0}}\delta Q\right),\;l_{2}\approx l_{⊥}^{0}\left(1+\frac{l_{⊥,Q}}{l_{⊥}^{0}}\delta Q+\frac{l_{2,b}}{l_{⊥}^{0}}b\right),\;l_{3}\approx l_{⊥}^{0}\left(1+\frac{l_{⊥,Q}}{l_{⊥}^{0}}\delta Q+\frac{l_{3,b}}{l_{⊥}^{0}}b\right),$$

where $l_{∥,Q}=\partial l_{1}/\partial Q$ and *l*_⊥,*Q*_ = ∂*l*_2_/∂*Q* = ∂*l*_3_/∂*Q* denote the first derivatives of *l*_1_ and *l*_2_ (and also *l*_3_) with respect to *Q* at *b* = 0, respectively, and *l*_2,*b*_ = ∂*l*_2_/∂*b* and *l*_3,*b*_ = ∂*l*_3_/∂*b* are the first derivatives of *l*_2_ and *l*_3_, respectively, with respect to *b* at *Q* = *Q*_0_.

For a nematic solid with the strain-energy density given by (5.6), we derive the stretch and shear moduli in a direction parallel or perpendicular to the nematic director, as follows.

### (a) Extension parallel to the director

When the nematic director is uniformly aligned in the first direction, we take $\bar{\text{F}}=\mathrm{diag}(\lambda_{1},\lambda_{2},\lambda_{3})$, where *λ*_1_ = *λ* > 1 and $\lambda_{2}=\lambda_{3}=1/\sqrt{\lambda }$. Assuming infinitesimal extension, we have *λ* = 1 + ε and *λ*^−1^ = 1 − ε, where ε denotes the infinitesimal strain. At *b* = 0, the elastic strain-energy density is approximated by

5.8 $$\begin{array}{r} {\tilde{W}}^{\left(\text{nc}\right)}\approx \frac{\mu }{2}(\lambda^{2}+2\lambda^{-1})-\frac{\mu }{2}\left(\lambda^{2}\frac{l_{∥,Q}}{l_{∥}^{0}}+2\lambda^{-1}\frac{l_{⊥,Q}}{l_{⊥}^{0}}\right)\delta Q. \end{array}$$

This contributes to the small variation in total strain-energy density defined by (5.6)

5.9 $$\begin{array}{r} \delta {\tilde{W}}^{\left(\text{lce}\right)}=\frac{3\mu }{2}\varepsilon^{2}-\mu \left(\frac{l_{∥,Q}}{l_{∥}^{0}}-\frac{l_{⊥,Q}}{l_{⊥}^{0}}\right)\varepsilon \delta Q+\frac{1}{2}{\tilde{W}}_{QQ}^{\left(\text{lce}\right)}{\left(\delta Q\right)}^{2}, \end{array}$$

where ${\tilde{W}}_{QQ}^{\left(\text{lce}\right)}=\partial^{2}{\tilde{W}}^{\left(\text{lce}\right)}/\partial Q^{2}{|}_{Q=Q_{0}}$. The above quadratic function in *δQ* has a minimum of

5.10 $$\min_{\delta Q}\delta {\tilde{W}}^{\left(\text{lce}\right)}=\frac{3\mu }{2}\varepsilon^{2}\left[1-\frac{\mu }{3{\tilde{W}}_{QQ}^{\left(\text{lce}\right)}}{\left(\frac{l_{∥,Q}}{l_{∥}^{0}}-\frac{l_{⊥,Q}}{l_{⊥}^{0}}\right)}^{2}\right].$$

Denoting *E* = 3*μ*, the stretch modulus in a direction parallel to the nematic director is then

5.11 $$E_{∥}=E\left[1-\frac{\mu }{3{\tilde{W}}_{QQ}^{\left(\text{lce}\right)}}{\left(\frac{l_{∥,Q}}{l_{∥}^{0}}-\frac{l_{⊥,Q}}{l_{⊥}^{0}}\right)}^{2}\right].$$

### (b) Extension perpendicular to the director

When the director is aligned in the first direction and $\bar{\text{F}}=\mathrm{diag}(\lambda_{1},\lambda_{2},\lambda_{3})$, where $\lambda_{1}=\lambda_{3}=1/\sqrt{\lambda }$ and *λ*_2_ = *λ* > 1, assuming *λ* = 1 + ε and *λ*^−1^ = 1 − ε, with ε the infinitesimal strain, the elastic strain-energy density given by (5.4) is approximated by

5.12 $${\tilde{W}}^{\left(\text{nc}\right)}\approx \frac{\mu }{2}(\lambda^{2}+2\lambda^{-1})-\frac{\mu }{2}\left(\lambda^{-1}\frac{l_{∥,Q}}{l_{∥}^{0}}+\lambda^{2}\frac{l_{⊥,Q}}{l_{⊥}^{0}}+\lambda^{-1}\frac{l_{⊥,Q}}{l_{⊥}^{0}}\right)\delta Q-\frac{\mu }{2}\left(\lambda^{2}\frac{l_{2,b}}{l_{⊥}^{0}}+\lambda^{-1}\frac{l_{3,b}}{l_{⊥}^{0}}\right)b.$$

This contributes to the total strain-energy variation

5.13 $$\delta {\tilde{W}}^{\left(\text{lce}\right)}=\frac{3\mu }{2}\varepsilon^{2}+\frac{\mu }{2}\left(\frac{l_{∥,Q}}{l_{∥}^{0}}-\frac{l_{⊥,Q}}{l_{⊥}^{0}}\right)\varepsilon \delta Q-\frac{\mu }{2}\left(2\frac{l_{2,b}}{l_{⊥}^{0}}-\frac{l_{3,b}}{l_{⊥}^{0}}\right)\varepsilon b+\frac{1}{2}{\tilde{W}}_{QQ}^{\left(\text{lce}\right)}{\left(\delta Q\right)}^{2}+\frac{1}{2}{\tilde{W}}_{bb}^{\left(\text{lce}\right)}b^{2},$$

where ${\tilde{W}}_{QQ}^{\left(\text{lce}\right)}$ is defined as before and ${\tilde{W}}_{bb}^{\left(\text{lce}\right)}=\partial^{2}{\tilde{W}}^{\left(\text{lce}\right)}/\partial b^{2}{|}_{b=0}$. Minimizing the above function with respect to *δQ* and *b* gives

5.14 $$\min_{\left(\delta Q,b\right)}\delta {\tilde{W}}^{\left(\text{lce}\right)}=\frac{3\mu }{2}\varepsilon^{2}\left[1-\frac{\mu }{12{\tilde{W}}_{QQ}^{\left(\text{lce}\right)}}{\left(\frac{l_{∥,Q}}{l_{∥}^{0}}-\frac{l_{⊥,Q}}{l_{⊥}^{0}}\right)}^{2}-\frac{\mu }{12{\tilde{W}}_{bb}^{\left(\text{lce}\right)}}{\left(2\frac{l_{2,b}}{l_{⊥}^{0}}-\frac{l_{3,b}}{l_{⊥}^{0}}\right)}^{2}\right].$$

Denoting *E* = 3*μ*, the stretch modulus in a direction perpendicular to the nematic director is

5.15 $$E_{⊥}=E\left[1-\frac{\mu }{12{\tilde{W}}_{QQ}^{\left(\text{lce}\right)}}{\left(\frac{l_{∥,Q}}{l_{∥}^{0}}-\frac{l_{⊥,Q}}{l_{⊥}^{0}}\right)}^{2}-\frac{\mu }{12{\tilde{W}}_{bb}^{\left(\text{lce}\right)}}{\left(2\frac{l_{2,b}}{l_{⊥}^{0}}-\frac{l_{3,b}}{l_{⊥}^{0}}\right)}^{2}\right].$$

### (c) Shear parallel to the director

We recall that a simple shear deformation of a hyperelastic material is equivalent to a biaxial stretch (‘pure shear’ [81]) in the principal directions [82]. We assume that the director is aligned uniformly in the first (or second direction), and $\bar{\text{F}}=\text{R}\;\mathrm{diag}(\lambda_{1},\lambda_{2},\lambda_{3})$, where *λ*_1_ = *λ* > 1, *λ*_2_ = 1/*λ* and *λ*_3_ = 1, and **R** is the rotation by an angle of *π*/4 in the plane formed by the first two directions. Taking *λ* = 1 + ε and *λ*^−1^ = 1 − ε, with ε the infinitesimal strain, the elastic strain-energy density is approximated as follows:

5.16 $$\begin{array}{rl} {\tilde{W}}^{\left(\text{nc}\right)} & =\frac{\mu }{4}\left[\lambda^{2}\left(\frac{l_{∥}^{0}}{l_{⊥}^{0}}+1\right)+\lambda^{-2}\left(\frac{l_{⊥}^{0}}{l_{∥}^{0}}+1\right)+2\right]\\ & \;-\frac{\mu }{4}\left[\lambda^{2}\frac{l_{∥,Q}}{l_{∥}^{0}}\left(\frac{l_{∥}^{0}}{l_{⊥}^{0}}+1\right)+\lambda^{-2}\frac{l_{⊥,Q}}{l_{⊥}^{0}}\left(\frac{l_{⊥}^{0}}{l_{∥}^{0}}+1\right)\right]\delta Q-\frac{\mu }{4}\frac{l_{2,b}}{l_{⊥}^{0}}\left(\lambda^{2}\frac{l_{∥}^{0}}{l_{⊥}^{0}}+\lambda^{-2}\right)b. \end{array}$$

This contributes to the total strain-energy variation

5.17 $$\begin{array}{rl} \delta {\tilde{W}}^{\left(\text{lce}\right)} & =\frac{\mu }{4}\varepsilon^{2}\left(\frac{l_{∥}^{0}}{l_{⊥}^{0}}+\frac{l_{⊥}^{0}}{l_{∥}^{0}}+2\right)-\frac{\mu }{2}\left[\frac{l_{∥,Q}}{l_{∥}^{0}}\left(\frac{l_{∥}^{0}}{l_{⊥}^{0}}+1\right)-\frac{l_{⊥,Q}}{l_{⊥}^{0}}\left(\frac{l_{⊥}^{0}}{l_{∥}^{0}}+1\right)\right]\varepsilon \delta Q\\ & \;-\frac{\mu }{2}\frac{l_{2,b}}{l_{⊥}^{0}}\left(\frac{l_{∥}^{0}}{l_{⊥}^{0}}-1\right)\varepsilon b+\frac{1}{2}{\tilde{W}}_{QQ}^{\left(\text{lce}\right)}{\left(\delta Q\right)}^{2}+\frac{1}{2}{\tilde{W}}_{bb}^{\left(\text{lce}\right)}b^{2}, \end{array}$$

with ${\tilde{W}}_{QQ}^{\left(\text{lce}\right)}$ and ${\tilde{W}}_{bb}^{\left(\text{lce}\right)}$ defined as before. We minimize this function with respect to *δQ* and *b*,

5.18 $$\begin{array}{rl} \min_{\left(\delta Q,b\right)}\delta {\tilde{W}}^{\left(\text{lce}\right)} & =\frac{\mu }{4}\varepsilon^{2}\{\left(\frac{l_{∥}^{0}}{l_{⊥}^{0}}+\frac{l_{⊥}^{0}}{l_{∥}^{0}}+2\right)-\frac{\mu }{2{\tilde{W}}_{QQ}^{\left(\text{lce}\right)}}{\left[\frac{l_{∥,Q}}{l_{∥}^{0}}\left(\frac{l_{∥}^{0}}{l_{⊥}^{0}}+1\right)-\frac{l_{⊥,Q}}{l_{⊥}^{0}}\left(\frac{l_{⊥}^{0}}{l_{∥}^{0}}+1\right)\right]}^{2}\\ & \;-\frac{\mu }{2{\tilde{W}}_{bb}^{\left(\text{lce}\right)}}{\left(\frac{l_{2,b}}{l_{⊥}^{0}}\right)}^{2}{\left(\frac{l_{∥}^{0}}{l_{⊥}^{0}}-1\right)}^{2}\}. \end{array}$$

The linear shear modulus in a direction parallel to the nematic director is then

5.19 $$\begin{array}{rl} \mu_{∥} & =\frac{\mu }{4}\{\left(\frac{l_{∥}^{0}}{l_{⊥}^{0}}+\frac{l_{⊥}^{0}}{l_{∥}^{0}}+2\right)-\frac{\mu }{2{\tilde{W}}_{QQ}^{\left(\text{lce}\right)}}{\left[\frac{l_{∥,Q}}{l_{∥}^{0}}\left(\frac{l_{∥}^{0}}{l_{⊥}^{0}}+1\right)-\frac{l_{⊥,Q}}{l_{⊥}^{0}}\left(\frac{l_{⊥}^{0}}{l_{∥}^{0}}+1\right)\right]}^{2}\\ & \;-\frac{\mu }{2{\tilde{W}}_{bb}^{\left(\text{lce}\right)}}{\left(\frac{l_{2,b}}{l_{⊥}^{0}}\right)}^{2}{\left(\frac{l_{∥}^{0}}{l_{⊥}^{0}}-1\right)}^{2}\}. \end{array}$$

### (d) Shear perpendicular to the director

Next, assuming that the director is aligned in the first direction, we set $\bar{\text{F}}=\mathrm{diag}(\lambda_{1},\lambda_{2},\lambda_{3})$, where *λ*_1_ = 1, *λ*_2_ = *λ* > 1 and *λ*_3_ = 1/*λ*. Taking *λ* = 1 + ε and *λ*^−1^ = 1 − ε, with ε the infinitesimal strain, the elastic strain-energy density is approximated by

5.20 $${\tilde{W}}^{\left(\text{nc}\right)}\approx \frac{\mu }{2}(1+\lambda^{2}+\lambda^{-2})-\frac{\mu }{2}\left(\frac{l_{∥,Q}}{l_{∥}^{0}}+\lambda^{2}\frac{l_{⊥,Q}}{l_{⊥}^{0}}+\lambda^{-2}\frac{l_{⊥,Q}}{l_{⊥}^{0}}\right)\delta Q-\frac{\mu }{2}\left(\lambda^{2}\frac{l_{2,b}}{l_{⊥}^{0}}+\lambda^{-2}\frac{l_{3,b}}{l_{⊥}^{0}}\right)b.$$

This contributes to the small variation in total strain-energy density

5.21 $$\delta {\tilde{W}}^{\left(\text{lce}\right)}=\mu \varepsilon^{2}-\mu \left(\frac{l_{2,b}}{l_{⊥}^{0}}-\frac{l_{3,b}}{l_{⊥}^{0}}\right)\varepsilon b+\frac{1}{2}{\tilde{W}}_{bb}^{\left(\text{lce}\right)}b^{2},$$

where ${\tilde{W}}_{bb}^{\left(\text{lce}\right)}$ is defined as before. The above quadratic function in *b* has a minimum value of

5.22 $$\min_{b}\delta {\tilde{W}}^{\left(\text{lce}\right)}=\mu \varepsilon^{2}\left[1-\frac{\mu }{2{\tilde{W}}_{bb}^{\left(\text{lce}\right)}}{\left(\frac{l_{2,b}}{l_{⊥}^{0}}-\frac{l_{3,b}}{l_{⊥}^{0}}\right)}^{2}\right].$$

The linear shear modulus in a direction perpendicular to the nematic director is equal to

5.23 $$\mu_{⊥}=\mu \left[1-\frac{\mu }{2{\tilde{W}}_{bb}^{\left(\text{lce}\right)}}{\left(\frac{l_{2,b}}{l_{⊥}^{0}}-\frac{l_{3,b}}{l_{⊥}^{0}}\right)}^{2}\right].$$

### (e) Deviation from isotropy

The stretch and shear moduli $E_{∥}$, *E*_⊥_, $\mu_{∥}$ and *μ*_⊥_, defined by (5.11), (5.15), (5.19) and (5.23), respectively, contain information about the nematic order, in addition to the small strain elasticity of the polymer matrix, described by Young’s modulus *E* and shear modulus *μ*. Proceeding as in §§3 and 4, with ${\tilde{W}}^{\left(\text{lce}\right)}$ instead of *W*^(nc)^, by replacing *E* with $E_{∥}$ in (3.8) and with *E*_⊥_ in (3.9), *μ* with $\mu_{∥}$ in (4.7) and (4.10), and with *μ*_⊥_ in (4.13), we obtain

5.24 $$\frac{E^{\left(2\right)}}{E^{\left(1\right)}}=\frac{E_{⊥}}{E_{∥}}r^{2},\;\frac{\mu^{\left(2\right)}}{\mu^{\left(1\right)}}=r,\;\frac{\mu^{\left(3\right)}}{\mu^{\left(2\right)}}=\frac{\mu_{⊥}}{\mu_{∥}}r.$$

We deduce that: if $E_{∥}/E_{⊥}>1$, then *E*^(2)^/*E*^(1)^ < *r*^2^; if $E_{∥}/E_{⊥}<1$, then *E*^(2)^/*E*^(1)^ > *r*^2^; if $\mu_{∥}/\mu_{⊥}>1$, then *μ*^(3)^/*μ*^(2)^ < *r*; if $\mu_{∥}/\mu_{⊥}<1$, then *μ*^(3)^/*μ*^(2)^ > *r*. In particular, when *r* > 1, if $E_{∥}/E_{⊥}>r^{2}>1$ and $\mu_{∥}/\mu_{⊥}<1$, then *E*^(1)^ > *E*^(2)^ and *μ*^(1)^ < *μ*^(2)^ < *μ*^(3)^, which is qualitatively different from the behaviour of ideal nematic solids or any standard anisotropic hyperelastic material. In the next section, we show how to access these moduli through molecular dynamics simulations.

## 6. Molecular dynamics simulation

We performed molecular dynamics (MD) simulations to synthesize LCEs, form nematic LCEs and characterize their response under stretch and shear deformations. Given its accuracy and efficiency for modelling mesogen–polymer systems, we used a hybrid force field, including Gay–Berne coarse-graining potentials for mesogen–mesogen interaction and Lennard-Jones (LJ) potentials for united atoms of hydrocarbon groups, CH_*x*_, in polymer chains. Our computer simulations [83] can serve as a virtual experiment to observe the macroscopic mechanical behaviour, which can then be compared directly with the continuum theory, and to calculate the physical quantities arising from atomistic movement and gain a mechanistic understanding.

In the MD simulations of LCEs, the potential energy of the whole system includes contributions from bond stretch, angle bending, dihedral rotation, non-bonded LJ interaction between united atoms (*a*-*a*), anisotropic non-bonded Gay–Berne interaction between mesogens (*m*-*m*) and the extended Gay–Berne interaction between united atoms and mesogens (*a*-*m*):

6.1*a* $$E=E_{\text{bond}}+E_{\text{angle}}+E_{\text{dihedral}}+E_{a\text{-}a}+E_{m\text{-}m}+E_{a\text{-}m},$$

where

6.1*b* $$\begin{array}{rl} & E_{\text{bond}}=\sum_{i=1}^{N_{b}^{\left(1\right)}}\frac{k_{b}}{2}{\left(l_{i}-l^{\left(1\right)}\right)}^{2}+\sum_{i=1}^{N_{b}^{\left(2\right)}}\frac{k_{b}}{2}{\left(l_{i}-l^{\left(2\right)}\right)}^{2}, \end{array}$$

6.1*c* $$\begin{array}{rl} & E_{\text{angle}}=\sum_{i=1}^{N_{a}^{\left(1\right)}}\frac{k_{a}}{2}{\left(\theta_{i}-\theta^{\left(1\right)}\right)}^{2}+\sum_{i=1}^{N_{a}^{\left(2\right)}}\frac{k_{a}}{2}{\left(\theta_{i}-\theta^{\left(2\right)}\right)}^{2}, \end{array}$$

6.1*d* $$\begin{array}{rl} & E_{\text{dihedral}}=\sum_{i=1}^{N_{t}^{\left(1\right)}}\sum_{n=1}^{3}C_{n-1}^{\left(1\right)}{\left(\cos\phi_{i}\right)}^{n}+\sum_{i=1}^{N_{t}^{\left(2\right)}}\sum_{n=1}^{3}C_{n-1}^{\left(2\right)}{\left(\cos\phi_{i}\right)}^{n}, \end{array}$$

6.1*e* $$\begin{array}{rl} & E_{a\text{-}a}=\sum_{i=1}^{N_{ij}^{\left(a\text{-}a\right)}}\left[\frac{a_{i}a_{j}}{r_{ij}^{12}}-\frac{c_{i}c_{j}}{r_{ij}^{6}}\right] \end{array}$$

6.1*f* $$\begin{array}{rl} \;\text{and}\; & E_{m\text{-}m}=\sum_{i=1}^{N_{ij}^{\left(m\text{-}m\right)}}U_{r}({\text{A}}_{i},{\text{A}}_{j},{\text{r}}_{ij},\gamma ,\epsilon ,\sigma )\cdot \eta ({\text{A}}_{i},{\text{A}}_{j},v)\cdot \chi ({\text{A}}_{i},{\text{A}}_{j},{\text{r}}_{ij},\xi ). \end{array}$$

**Figure 6.**
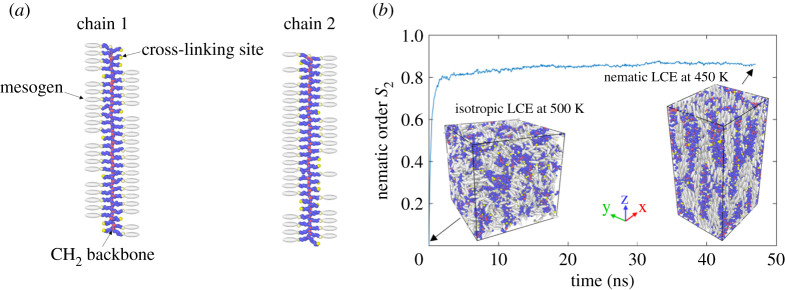
(*a*) Schematic of single chains of a liquid crystal polymer with a hydrocarbon backbone and 50 side chains among which 20% are attached with cross-linking sites (yellow atoms) and 80% are attached with mesogens (white ellipsoids). The cross-linking sites and mesogens are randomly selected from the 50 side chains for each liquid crystal polymer molecule and chain 1 and chain 2 are displayed here as examples. (*b*) The evolution of nematic order *S*_2_ during the isotropic–nematic phase transition when the weakly cross-linked LCEs were first quenched from 500 K to 450 K with external field for 10 ns, then equilibrated at 450 K with external field for 20 ns, then equilibrated at 450 K without external field for 17 ns. (Online version in colour.)

In the above equations, *E*_bond_ represents a harmonic bond style, with *l*_*i*_ the *i*th bond length, *l*^(1)^ the equilibrium bond length of CH_*x*_–CH_*y*_ and *l*^(2)^ the equilibrium bond length of CH_*x*_–mesogen; *E*_angle_ denotes a harmonic angle style, with *θ*_*i*_ the *i*th angle, *θ*^(1)^ the equilibrium angle of the non-branched X–*CH*_2_–*X* and *θ*^(2)^ the equilibrium angle of branched X–CH–X; *E*_dihedral_ is a multiple-term harmonic dihedral style, with *ϕ*_*i*_ the *i*th torsion angle, $C_{n}^{\left(1\right)}$ the non-branched X–CH_2_–CH_2_–X and $C_{n}^{\left(2\right)}$ the branched X–CH_2_–CH–X; *E*_(*a*–*a*)_ represents the LJ potential between non-bonded united atoms CH_*x*_, with *r*_*ij*_ the distance between two united atoms and *a*_*i*_, *c*_*i*_ the factorized energy parameters for CH_*x*_; *E*_*m*−*m*_ is the Gay–Berne potential between non-bonded mesogens, with *U*_*r*_ the shifted distance-dependent interaction, *η*, *χ* the orientation-dependent energy, **A**_*i*_ the transformation matrix for mesogen *i*, **r**_*ij*_ the centre-to-centre vector between the *i*th and *j*th mesogens and all the rest of the parameters specified as constants in table 1; and *E*_*a*−*m*_ denotes the extended Gay–Berne potential between a non-bonded mesogen and CH_*x*_ following the standard mixing rule [86]. The cut-off distance is 9.8 Å for the LJ potential and 16.8 Å for the Gay–Berne potential. For the detailed explanation and explicit form of the Gay–Berne potential, readers should refer to [87,88]. First, 64 molecules of side-chain liquid crystal polymers were created, where every molecule has a backbone of 100 hydrocarbon monomers and 50 side chains attaching to the backbone in a syndiotactic way. Among the 50 side chains for each molecule, 20% were randomly selected to be attached with cross-linking sites, and the rest were attached with mesogens, as shown in figure 6*a*. Thus, every LC molecule has a different configuration. The 64 molecules were mixed by heating at 800 K for 50 ns. Then the system was quenched down to 500 K during 10 ns. Cross-linking of the first step was established while equilibrating the system at 500 K. A weakly cross-linked isotropic LCE was constructed, as shown in figure 6*b*. The system was found to have its isotropic–nematic phase-transition temperature below 490 K, consistent with other MD studies of similar LCE systems [89]. To form nematic LCEs, the whole system was quenched down to 450 K with an external field $U_{i}^{\text{efield}}=-1.0\cdot P_{2}(\cos\theta_{i})$, where *P*_2_(*x*) = (3*x*^2^ − 1)/2 is the second Legendre polynomial and *θ*_*i*_ is the angle between the long axis of the *i*th mesogen and the external field. The external field has been experimentally and computationally proved to accelerate the formation of the nematic phase [90,91]. The quenching stage from 500 K to 450 K lasted 10 ns and the equilibrium stage at 450 K lasted 20 ns, for both of which the external field was applied along the *z*-direction. The external field was removed while the LCE system was equilibrated at 450 K for another 17 ns and the nematic phase was found to be stable. The nematic order *S*_2_ = 〈*P*_2_(cos*θ*_*i*_)〉 is shown in figure 6*b* during the quenching and equilibrium state. Subsequently, the second stage of cross-linking was performed within the nematic LCEs. The nematic system was then quenched down to 300 K and the nematic order was found to be well maintained. Throughout the whole process of constructing nematic LCEs, NPT calculations were performed using a time step of 1 fs in the Large-scale Atomic/Molecular Massively Parallel Simulator (LAMMPS) with periodic boundary conditions along three dimensions. At the isotropic–nematic transition, a spontaneous deformation was observed in the MD simulations shown in figure 6*b*, owing to the alignment of ellipsoidal mesogens along the *Z*-direction. The nematic LCE at 300 K has three dimensions: $l_{x}^{\mathrm{nc}}=99.6537\;\text{Å}$, $l_{y}^{\mathrm{nc}}=88.1515\;\text{Å}$ and $l_{z}^{\mathrm{nc}}=185.7362\;\text{Å}$. During the isotropic–nematic phase transition, the volume of LCEs should demonstrate a negligible change [92]. However, the MD simulations of LCEs using Gay–Berne potentials are known to show thermal expansion effects [93]. To eliminate these non-physical effects, the isotropic LCE at 500 K was quickly quenched down to 300 K and the three dimensions for the isotropic phase were measured as $l_{x}^{\text{iso}}=120.3505\;\text{Å}$, $l_{y}^{\text{iso}}=118.7118\;\text{Å}$ and $l_{z}^{\text{iso}}=117.2290\;\text{Å}$. The respective stretch ratios were then calculated as *λ*_*x*_ = 0.8280, *λ*_*y*_ = 0.7426 and *λ*_*z*_ = 1.5844. Ideally, the biaxial Gay–Berne potential for mesogen–mesogen interaction should yield the same stretch ratio along the *X*- and *Y*-directions. Here, however, the small size of the LCE system in the MD simulations gives rise to slightly different *λ*_*x*_ and *λ*_*y*_. To eliminate the size effect on the spontaneous deformation, these stretch ratios were averaged to *λ*_*x*−*y*_ = 0.7853. Then the anisotropy ratio can be estimated as *r* ≈ *λ*_*z*_/*λ*_*x*−*y*_ = 2.0176, Poisson’s ratio is *ν* ≈ 0.5 and *a* = *r*^2^ ≈ 4.0706.

**Table 1.** Parameters for interatomic potentials used in the MD simulation [84,85].

parameters	value (units)
*k*_*b*_, bond energy constant	520.0156 (kcal/mol/Å^2^)
*l*^(1)^, equilibrium CH_*x*_–*CH*_*y*_ bond length	1.540 (Å)
*l*^(2)^, equilibrium CH_*x*_–mesogen bond length	7.075 (Å)
*k*_*a*_, angle energy constant	124.2009 (kcal/mol)
*θ*^(1)^, equilibrium non-branched angle	114.0014 (°)
*θ*^(2)^, equilibrium branched angle	112.0018 (°)
$C_{0}^{\left(1\right)}$, non-branched torsion energy constant	2.0066 (kcal/mol)
$C_{0}^{\left(2\right)}$, non-branched torsion energy constant	4.0111 (kcal/mol)
$C_{0}^{\left(3\right)}$, non-branched torsion energy constant	0.2709 (kcal/mol)
$C_{0}^{\left(4\right)}$, non-branched torsion energy constant	−6.2885 (kcal/mol)
$C_{1}^{\left(1\right)}$, branched torsion energy constant	0.7413 (kcal/mol)
$C_{1}^{\left(2\right)}$, branched torsion energy constant	1.8264 (kcal/mol)
$C_{1}^{\left(3\right)}$, branched torsion energy constant	0.5329 (kcal/mol)
$C_{1}^{\left(4\right)}$, branched torsion energy constant	−3.4521 (kcal/mol)
*a*_*i*_, energy parameter for CH_3_	$2534.0341\;(\sqrt{\text{kcal/mol}}\cdot {\text{Å}}^{6})$
*c*_*i*_, energy parameter for CH_3_	$47.2921\;(\sqrt{\text{kcal/mol}}\cdot {\text{Å}}^{3})$
*a*_*i*_, energy parameter for CH_2_	$2251.9322\;(\sqrt{\text{kcal/mol}}\cdot {\text{Å}}^{6})$
*c*_*i*_, energy parameter for CH_2_	$37.0955\;(\sqrt{\text{kcal/mol}}\cdot {\text{Å}}^{3})$
*a*_*i*_, energy parameter for CH_1_	$1467.0522\;(\sqrt{\text{kcal/mol}}\cdot {\text{Å}}^{6})$
*c*_*i*_, energy parameter for CH_1_	$21.2860\;(\sqrt{\text{kcal/mol}}\cdot {\text{Å}}^{3})$
*ϵ*, well depth for *U*_*r*_ function in (6.1*a*–*f*)	0.8079 (kcal/mol)
*σ*, minimum effective particle radii in (6.1*a*–*f*)	5 (Å)
*ϵ*_*i*_, relative well depth for end-to-end	5
*ϵ*_*i*_, relative well depth for side-to-side	1
*v*, exponent for *η* function in (6.1*a*–*f*)	1
*ξ*, exponent for *χ* function in (6.1*a*–*f*)	2
*κ*, length/breadth ratio of mesogen	3
mass, united atoms CH_*x*_	12.0 + *x* (g/mol)
mass, mesogen	226.0 (g/mol)

**Figure 7.**
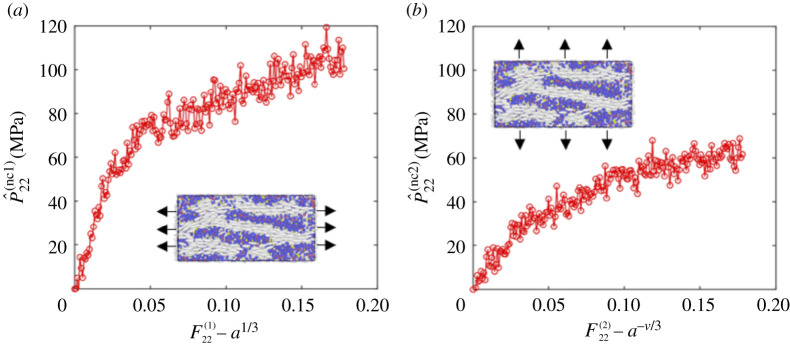
The first Piola–Kirchhoff tensile stress in the nematic LCE system when (*a*) the director is parallel to the tensile force and (*b*) the director is perpendicular to the tensile force. (Online version in colour.)

**Figure 8.**
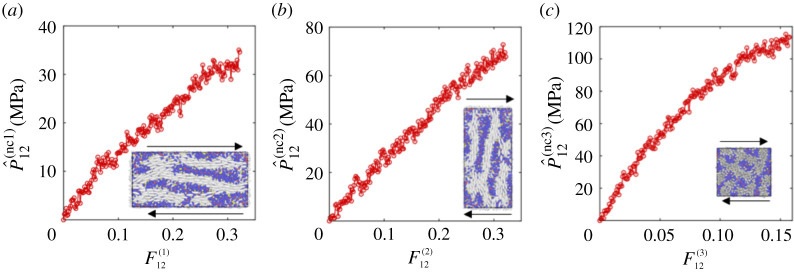
The first Piola–Kirchhoff shear stress in the nematic LCE system when (*a*) the director is parallel to the shear force, (*b*)the director is perpendicular to the shear force and (*c*) the director is perpendicular to the shear plane. (Online version in colour.)

After obtaining the nematic LCE at 300 K, we subjected the system to either stretch or shear deformations as described in §§3 and 4, respectively. For each deformation, we can calculate the evolution of the first Piola–Kirchhoff stresses with respect to the strains. We first calculate the Cauchy stress tensor in LAMMPS based on $T_{ij}=\Sigma_{k}^{N}m_{k}v_{ki}v_{kj}/V+\Sigma_{k}^{N^{\prime }}r_{ki}f_{kj}/V$, where *m*_*k*_ represents the mass of the *k*th atom, *v*_*ki*_ denotes the velocity of the *k*th atom in the *i*th dimension, *N* is the total number of atoms, *V* is the total volume of the system, *N*′ is the number of atoms pairs and *r*_*ki*_ and *f*_*kj*_ are the positions and forces of atom *k* along the *i*th and *j*th dimensions, respectively [94]. Next, given the deformation imposed, we construct the elastic deformation tensor **A** and, subsequently, the corresponding first Piola–Kirchhoff stress tensor **P**, following (2.6). Then, imposing the spontaneous deformation tensor **G**, or simply following (3.6), (3.7), (4.6), (4.9) or (4.12), we obtain the final ${\hat{P}}_{ij}^{\left(\text{nc}\right)}$. The results are shown in figures 7 and 8, respectively, where all the cases demonstrate some nonlinearity in the stress-deformation relations. From the two stretch deformations, we derive the effective stretch moduli *E*^(1)^ = 2253.4 MPa and *E*^(2)^ = 775.475 MPa. From the three shear deformations, we find the effective shear moduli *μ*^(1)^ = 132.78 MPa, *μ*^(2)^ = 258.26 MPa and *μ*^(3)^ = 889.97 MPa. Thus,

6.2 $$\frac{E^{\left(2\right)}}{E^{\left(1\right)}}=0.3441<r^{2},\;\frac{\mu^{\left(2\right)}}{\mu^{\left(1\right)}}=1.9450\approx r,\;\frac{\mu^{\left(3\right)}}{\mu^{\left(2\right)}}=3.4460>r.$$

These relations are in agreement with (5.24) when $E_{∥}/E_{⊥}>1$ and $\mu_{∥}/\mu_{⊥}<1$ (see also fig. 7 of [38], fig. 7 of [45], fig. 6 of [48] and fig. 3 of [51]). Our theoretical results are independent of the manufacturing history. Experimentally, the mechanical properties of main-chain and side-chain LCEs are compared in [51]. In future work, it would be interesting to carry out separate simulations for these different cases to compare their behaviour computationally as well.

## 7. Conclusion

We studied theoretically and computationally the mechanical behaviour of nematic LCEs under different stretch and shear deformations. Theoretically, we first examined ideal nematic elastomers characterized by a homogeneous isotropic elastic strain-energy density, then also phenomenological models incorporating an additional nematic energy. We showed that these cases are qualitatively different, and that the generalized model does not necessarily order stretch moduli in the same way as the shear moduli. We also performed molecular dynamics simulations to analyse numerically the responses of simulated systems under a similar set of deformations, and found different mechanical responses in different directions. Therefore, the trifecta of experiments, computations and theory leads us to conclude that the contribution of the nematic free energy cannot be ignored, even in small deformations, and that LCEs are best understood as aeolotropic materials. When Frank effects also play an important role, they need to be taken into account as well. However, the deviation from isotropy is well captured by including the nematic energy, and this constitutes an important step in the constitutive modelling of liquid crystalline solids.

## Data accessibility

All data for this research are openly available at https://doi.org/10.5281/zenodo.5156815.

## Appendix A. Stresses in nematic solids

In this appendix, we formulate the stress tensors of a nematic material described by the strain-energy function given by (2.1) in terms of the stresses in the base polymeric network. These relations were originally presented in [65] and are provided here for convenience.

If the nematic director is ‘free’ to rotate relative to the elastic matrix, then **F** and **n** are independent variables, and the Cauchy stress tensor for the nematic material with the strain-energy function described by (2.1) is calculated as follows:

A 1 $${\text{T}}^{\left(\text{nc}\right)}=J^{-1}\frac{\partial W^{\left(\text{nc}\right)}}{\partial \text{F}}{\text{F}}^{T}-p^{\left(\text{nc}\right)}\text{I}=J^{-1}{\text{G}}^{-1}\frac{\partial W}{\partial \text{A}}{\text{A}}^{T}\text{G}-p^{\left(\text{nc}\right)}\text{I}=J^{-1}{\text{G}}^{-1}\text{T}\text{G},$$

where **T** is the Cauchy stress tensor defined by (2.5), J=detF and the scalar *p*^(nc)^ represents the Lagrange multiplier for the internal constraint *J* = 1. Then

A 2 $${\text{P}}^{\left(\text{nc}\right)}={\text{T}}^{\left(\text{nc}\right)}\mathrm{Cof}(\text{F})={\text{G}}^{-1}\text{T}{\text{A}}^{-T}={\text{G}}^{-1}\text{P}$$

is the first Piola–Kirchhoff stress tensor for the nematic solid material, with **P** the first Piola–Kirchhoff stress given by (2.6).

If the nematic director is ‘frozen’, the Cauchy stress tensor for the nematic material takes the form

A 3 $${\hat{\text{T}}}^{\left(\text{nc}\right)}=J^{-1}{\text{G}}^{-1}\text{T}\text{G}-J^{-1}q\left(\text{I}-\frac{\text{F}{\text{n}}_{0}⊗\text{F}{\text{n}}_{0}}{|\text{F}{\text{n}}_{0}{|}^{2}}\right)\text{n}⊗\frac{\text{F}{\text{n}}_{0}}{|\text{F}{\text{n}}_{0}|},$$

where **T** is the Cauchy stress defined by (2.5), J=detF, *p*^(nc)^ is the Lagrange multiplier for the volume constraint *J* = 1 and *q* is the Lagrange multiplier for the constraint (2.4). Then

A 4 $${\hat{\text{P}}}^{\left(\text{nc}\right)}={\hat{\text{T}}}^{\left(\text{nc}\right)}\mathrm{Cof}(\text{F})$$

is the first Piola–Kirchhoff stress tensor for the nematic solid.
